# Employment status, working conditions and depressive symptoms among German employees born in 1959 and 1965

**DOI:** 10.1007/s00420-014-0999-5

**Published:** 2014-11-22

**Authors:** Hermann Burr, Angela Rauch, Uwe Rose, Anita Tisch, Silke Tophoven

**Affiliations:** 1 Federal Institute for Occupational Safety and Health (BAuA), Nöldnerstraße 40-42, 10317 Berlin, Germany; 2 Institute for Employment Research (IAB), Regensburger Straße 104, 90478 Nuremberg, Germany

**Keywords:** Employment history, Employment status, Depressive symptoms, Job insecurity, Leadership quality, Influence at work

## Abstract

**Purpose:**

We investigated whether (1) current employment status (regular full-time, regular part-time and marginal employment) is associated with depressive symptoms and (2) whether these associations are mediated by current working conditions and previous employment history.

**Methods:**

Two cohorts of German employees aged 46 and 52 years were selected from administrative data of the German Federal Employment Agency and answered questions about depressive symptoms (we use an applied version of BDI-V) and their current working conditions. In addition, the participants gave written consent to link register data regarding their employment histories (*n* = 4,207). Multiple linear regression analyses were conducted.

**Results:**

Men experienced elevated depressive symptoms when working regular part-time; women experienced such symptoms when engaged in marginal employment. These associations decreased when we adjusted for job insecurity and rose slightly when we adjusted for leadership quality. Men and women who reported a low level of influence at work showed a higher risk of depressive symptoms. For women, the association between current employment position and depressive symptoms could be partly explained by low levels of influence at work. For men, the association between depressive symptoms and current regular part-time employment decreased when we adjusted for previous part-time employment. Conversely, for women, the association with depressive symptoms increased in current regular part-time and marginal employment when we adjusted for employment history.

**Conclusions:**

In both genders, the observed associations between depressive symptoms and current employment status were mediated by both current psychosocial conditions and employment history. Employees not having a regular full-time job differed from full-time employees with respect to both their current working conditions and their employment history.

## Aim

This study aimed to investigate whether current employment status (regular full-time, regular part-time or marginal employment) is associated with depressive symptoms among German employees. It is essential to analyse potential occupational risk factors for the development of depressive symptoms, because mental disorders such as depressive symptoms are the main reason for disability pension in Germany (German Federal Pension Insurance 2012); they have also been found longitudinally to predict disability pensioning (Bültmann 2008). In the present study, marginal employment comprised (1) minor employment [with comparatively low income and reduced social security contributions (Eichhorst and Marx 2011)], (2) job creation schemes and (3) employment of a minimum of at least one hour per week held by people who were not employed, e.g. sick-listed, unemployed, etc. In contrast, regular full-time and part-time employment is characterised by a higher level of social protection. More specifically, we investigate (1) whether current part-time or marginal employment compared to regular full-time employment was associated with depressive symptoms among middle-aged German employees, and (2) to what extent this association was related to current working conditions and previous employment history. Depressive symptoms included emotional issues such as depressed mood, diminished pleasure, insomnia or hypersomnia, loss of energy and diminished ability to think or recurrent suicidal ideations (American Psychiatric Association 2000, 2013).

In Germany, most middle-aged employees work in full-time or part-time employment with a fair level of social protection (Hasselhorn et al. 2014; Tisch and Tophoven 2012). However, the prevalence of part-time work has increased substantially—e.g. from 19 % in 1999 to 26 % in 2009 (Sandor 2011). The increase in part-time employment is related to the growing share of female workers—a trend which can be seen in many other continental European countries (Bosch 2004; Pfau-Effinger 1993). Among men, regular full-time employment remains the standard employment form. One cross-sectional study (Thielen and Kroll 2013) and one follow-up study (Wahrendorf et al. 2013) have previously considered the possible association between current employment status and depressive symptoms in Germany (the latter study pooled participants from Germany with participants in 11 other European countries). Both studies showed an increased risk of depressive symptoms among individuals who were not employed and among men engaged in minor employment. The follow-up study found that depressive symptoms among older men were associated with previous involuntary job loss, job instability and fragmented careers. These associations were not present among women.

At least two reasons for the association between part-time or marginal employment and depressive symptoms can be anticipated. First, it can be anticipated that the current employment status is part of an employment trajectory (Benach et al. 2014; Vosko 2006) which is thought to negatively affect health at advanced working age (Kessing et al. 2003; Montgomery et al. 1996, 1999; Mossakowski 2009; Wahrendorf et al. 2013), especially in the case of highly fragmented employment trajectories (Sirviö et al. 2012). Second, it can be argued that marginal employment on average is characterised by poorer working conditions (Sandor 2011; Scott-Marshall and Tompa 2011), which could negatively affect individual health.

To control for poor working conditions, we included three working conditions in our analyses that occur more often in marginal and part-time employment (Sandor 2011) and that predict depressive symptoms (Rugulies et al. 2008). Low levels of influence at work and low levels of social support from management predict the incidence of depressive symptoms (Magnusson Hanson et al. 2009; Rugulies et al. 2008; Siegrist et al. 2012). In addition, low levels of employment security threaten a worker’s psychological well-being and are a risk factor for depressive symptoms (Rugulies et al. 2008; Sirviö et al. 2012; Virtanen et al. 2002; Wahrendorf et al. 2013).

Depressive symptoms also might increase individuals’ risk of obtaining part-time or marginal employment (Pacheco et al. 2014), the risk of unemployment or the risk of disability pension (Bültmann et al. 2006). One reason might be that individuals with depressive symptoms have lower chances of completing vocational education (Giver et al. 2011) and in turn obtain less favourable employment positions (Blossfeld 1986). Additionally, it is reasonable that individuals with depressive symptoms see themselves obliged to work fewer hours because of their mental condition (Mechanic et al. 2002). Therefore, the relationship between employment status and health could be attributed either to the employment situation causing poor health (the causation hypothesis) or to the selection of people with poor health into unfavourable employment positions (the selection hypothesis) (Dahl 1996; Goldman 1994). In an analysis of the association between general health and labour market participation, Elstad and Krokstad (2003) found evidence for both causation and selection.

**Fig. 1:**
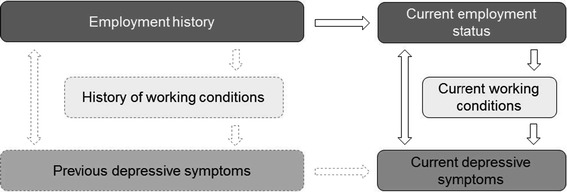
Conceptual model of the relationship between depressive symptoms, employment status, employment history and working conditions. *Boxes* and *arrows* in *broken lines* are not covered by the research question

For the following analyses, we set up a conceptual model (Fig. 1). As shown in the conceptual model, we assumed that current depressive symptoms are related to previous employment history. Although it cannot be tested with this current study, we further assume that previous depressive symptoms may have influenced employment trajectory. Moreover, the conceptual model considers the relationship between current employment and depressive symptoms, directly or indirectly mediated by working conditions. Finally, the model implies that current depressive symptoms can be related to depressive symptoms status in the past.

In the present paper, we are interested in the association between the current employment situation and depressive symptoms. First, we ask whether part-time or marginally employed individuals are disadvantaged regarding their risk to develop depressive symptoms in comparison with individuals in regular full-time employment. This question refers to the right column of the conceptual model. Second, we asked to what extent the association between the employment situation and depressive symptoms is mediated by current working conditions and individual employment history. This question is accounted for in the top row of the model. Due to data restrictions, we could not control for previous health conditions. This might have led to a selection problem, which we address in greater detail in the ‘Discussion’ section.

## Methods

### Population

To analyse the association between current employment status and depressive symptoms, we used data from the first wave of the lidA study (‘German cohort study on work, age and health’). Within lidA, a sample of two birth cohorts of German employees subject to social security contributions [including minor employment (Eichhorst and Marx 2011)] as of 31 December 2009 was selected from Integrated Employment Biography (IEB) register data of the German Federal Employment Agency (Dorner et al. 2010).^1^ In 2011, 6,585 personal interviews were conducted (i.e. the response rate was 27 %) (Hasselhorn et al. 2014). Selectivity analyses showed only minor differences between the lidA participants and the IEB sample, with a slightly lower probability of participation related to: (1) birth year (1965), (2) region (i.e. living in smaller cities or rural environments), (3) whether an individual was non-German and (4) whether a person had lower levels of qualification (Hasselhorn et al. 2014). At the time of the interview, 6,339 respondents were employed and 246 respondents were not. Of the employees, 4,921 respondents (78 %) gave written consent to link administrative records of their previous employment history to the survey data. Of those, 4,081 employed participants conducted a paper-and-pencil questionnaire on depressive symptoms [applied version of Beck Depression Inventory—BDI-V (Schmitt et al. 2003)] and answered questions on their current working conditions. Ultimately, 4,081 respondents are included in this paper’s analysis. Selected characteristics of our sample population can be found in Table 1.

**Table 1:** Sample characteristics (2011) (total *n* = 4,081; 1,933 men and 2,148 women)

	Men	Women
Age		
Born 1959	821 (42)	937 (44)
Born 1965	1,112 (58)	1,211 (56)
Education		
CASMIN low	66 (3)	110 (5)
CASMIN middle	1,392 (72)	1,631 (76)
CASMIN high	475 (25)	407 (19)
Partnership status		
In marriage or cohabitation	1,713 (89)	1,836 (85)
Current employment status		
Full-time	1,834 (95)	945 (44)
Part-time	62 (3)	1,011 (47)
Marginal	37 (2)	192 (9)
Working conditions		
Job insecurity^a^	207 (11)	209 (10)
Quality of leadership^b^	3.1 ± 0.9 (1–5)	3.2 ± 0.9 (1–5)
Influence at work^c^	2.7 ± 1.1 (1–5)	2.4 ± 1.1 (1–5)
Employment history		
Mostly full-time	1,778 (92)	925 (43)
Mostly part-time	52 (3)	771 (36)
Mostly other	103 (5)	452 (21)
Depressive symptoms (BDI-V)	17.9 ± 12.8 (0–91)	21.9 ± 14.0 (0–89)

Values are *n* (%) or mean ± SD (range)

^a^Absolute numbers (%)

^b^Range of answers [quality of leadership ranged between 5 (high quality of leadership) and 1 (low quality of leadership)]

^c^Range of answers [influence at work ranged between 5 (high influence at work) and 1 (low influence at work)]

The younger cohort, born in 1965, was oversampled in the first wave of lidA and represented approximately 57 % of the population studied. The reason for the oversampling was the expectation that in the following waves we would lose a considerable number of respondents of both cohorts for panel attrition reasons. In wave three (planned), the younger cohort is about the same age as the older cohort in wave one. To have approximately the same number of respondents of each cohort at each age, we included a higher amount of individuals of the younger cohort in the first wave. Due to gender-specific response rates, women made up 53 % of the study population. Both cohorts belonged to the German so-called baby boom generation and could be considered experienced by the time of this study (Tisch and Tophoven 2012). They showed similar employment trajectories and average levels of depressive symptoms. Intra-generational differences between these two cohorts were negligible.

A total of 22 % of the respondents held a university degree or an equivalent, approximately three-quarters of the respondents reported a medium level of secondary education, and fewer than 5 % of the respondents had never received a professional education. Almost nine in ten men and women were either married or cohabitating. With respect to current employment status, men predominantly held full-time employment (95 %), whereas women were more likely to work part-time (47 %) or in marginal employment (9 %) (cf. Table 1).

### Variables

*Depressive symptoms* were the dependent variable in our analyses. Depressive symptoms were assessed using the applied version of BDI-V, which covered all 20 items of the original clinical questionnaire except for weight loss (Schmitt et al. 2003). During the personal interviews, to avoid both possible interviewer effects and social desirability effects, the respondents were asked to fill in a paper-and-pencil questionnaire containing the variables of the BDI-V. Answers were summed to a score with values from 0 to 100. The inter-item correlations ranged from 0.21 to 0.68; Cronbach’s alpha was 0.92.

C*urrent employment status* was divided into three categories: regular full-time employment (at least 35 h per week), regular part-time employment and marginal employment (minor employment, job creation schemes and employment of a minimum of at least one hour per week held by people whose main status was not employed, e.g. sick-listed, unemployed, etc.).

*Influence at work* (i.e. decision authority) was measured by means of the following questions of the second version of the Copenhagen Psychosocial Questionnaire (COPSOQ II) (Pejtersen et al. 2010): ‘Do you have a say in choosing who you work with?’; ‘Can you influence the amount of work assigned to you?’; and ‘Do you have any influence on what you do at work?’. The response options (and values for the scale) were ‘Always’ (5); ‘Often’ (4); ‘Sometimes’ (3); ‘Seldom’ (2); and ‘Never/hardly ever’ (1). Mean values were calculated to represent the scale. Cronbach’s alpha was 0.67, and inter-item correlations ranged between 0.37 and 0.42.

*Quality of leadership* was measured by means of the following COPSOQ II questions (Pejtersen et al. 2010): ‘To what extent would you say that your immediate superior …’: (1) ‘… makes sure that the individual member of staff has good development opportunities?’; (2) ‘… gives high priority to job satisfaction?’; (3) ‘… is good at work planning?’; and (4) ‘… is good at solving conflicts?’. The response options (and values for the scale) were: ‘To a very large extent’ (5); ‘To a large extent’ (4); ‘Somewhat’ (3); ‘To a small extent’ (2); and ‘To a very small extent’ (1). Mean values were calculated to represent the scale. Cronbach’s alpha was 0.87; inter-item correlations ranged between 0.58 and 0.70.

*Job insecurity* was measured by a single item from the Effort Reward Questionnaire (Siegrist et al. 2009). Individuals were asked whether they consider their job at risk with the response options ‘Yes’ (1) and ‘No’ (0).

*Employment history* was generated based on administrative data from the German Federal Employment Agency that includes information on all individual periods of employment subject to social security contributions and unemployment. Based on previous employment history, we identified individuals’ main employment status for the previous 10 years (2001–2010). Accordingly, we distinguished between three categories: ‘Mostly full-time employment’ (majority of days in regular full-time employment), ‘Mostly part-time employment’ (majority of days in regular part-time employment) and ‘Mostly other’ (majority of days in minor employment, without employment or in employment not subject to social security contributions that is not included in the administrative data, e.g. self-employed or employed as a civil servant). Among men, ‘mostly other’ employment histories were dominated by days without social security contributions; among women, minor employment was the most dominant status (cf. Table 2). Men and women in current full-time employment were also mostly employed full-time in the past (88 %). Similarly, most marginally employed individuals had not been in regular full-time or part-time employment for the last 10 years. More than half of the currently part-time employed men and women also showed long periods of past part-time employment (cf. Table 3).

**Table 2:** Average days of employment by category of previous main employment history between 2001 and 2010 (total *n* = 4,081)

Previous work history			
Men (*n* = 1,933)	Mostly full-time (*n* = 1,778)	Mostly part-time (*n* = 52)	Mostly other (*n* = 103)
	Mean (SD)	Mean (SD)	Mean (SD)
Average days			
Regular full-time	3,470 (390)	436 (558)	793 (630)
Regular part-time	25 (149)	2,802 (830)	221 (494)
Minor employment	5 (50)	53 (230)	272 (632)
Subsidised employment	28 (108)	87 (203)	661 (820)
Unemployment	63 (182)	115 (274)	765 (824)
Without social security contributions	61 (188)	160 (326)	940 (1,059)

Women (*n* = 2,148)	Mostly full-time (*n* = 925)	Mostly part-time (*n* = 771)	Mostly other (*n* = 452)
	Mean (SD)	Mean (SD)	Mean (SD)
Average days			
Regular full-time	3,167 (625)	284 (464)	302 (478)
Regular part-time	205 (405)	2,999 (694)	364 (508)
Minor employment	45 (186)	80 (249)	1,473 (1,271)
Subsidised employment	50 (157)	46 (161)	271 (561)
Unemployment	82 (204)	66 (180)	304 (532)
Without social security contributions	104 (241)	178 (343)	938 (975)

**Table 3:** Main employment history categories between 2001 and 2010 by current employment status 2011 (total *n* = 4,081)

Current employment status			
	Regular full-time [*n* (%)]	Regular part-time [*n* (%)]	Marginal [*n* (%)]
Employment history			
Mostly full-time	2,439 (88)	240 (22)	–^a^
Mostly part-time	206 (7)	599 (56)	–^a^
Mostly other	134 (5)	234 (22)	187 (82)

^a^The number of cases was too small (<20) to report due to German data protection requirements for social data

As further control variables, we first included *level of education,* which was classified according to the CASMIN scheme using the two questions ‘What is the highest school qualification you have obtained?’ and ‘What is the highest vocational qualification you have obtained?’ (König et al. 1988). We distinguished between the three categories of ‘low’, ‘middle’ and ‘high’. Other control variables were *gender*, *cohort affiliation* and *partnership status*, which were based on information that the respondents gave during the interview.

### Analyses

The analyses were conducted with the statistics software Stata 12.0. Using survey set commands, we considered the sample point structure of the data set. To test our assumptions, we used a multiple linear regression analysis on depressive symptoms (BDI-V). First, we estimated bivariate regression models to see the uncontrolled contribution of each independent variable. In multivariate analyses, we first considered the subjects’ current employment contracts as an independent variable. In four additional regression analyses, we additionally controlled for current working conditions to see whether these mediated the relationship between current employment and depressive symptoms. First, we adjusted for job insecurity. Second, we considered leadership quality. Third, we considered influence in the workplace. Fourth, we considered the influence of the subjects’ previous employment histories in order to see whether they mediated the relationship between current employment and depressive symptoms. Finally, we included all tested variables and concepts in one analysis. At every step of the multivariate analyses, we further controlled for birth cohort and occupational education (CASMIN).

The assumption of normality was examined by the inspection of residual plots. A deviation was not detected. We also inspected possible multicollinearity for the intended regressions. Although we found that 88 % of men and women in regular full-time employment in 2011 had been full-time employed throughout the previous 10 years (2001–2010), the assumption of multicollinearity could be rejected due to low variance inflation factors (all values for VIF were below 1.7).

To identify gender differences in the influence of *job insecurity*, *quality of leadership*, *influence at work/decision authority* and the *main* type of current employment status *during the previous* 10 *years* on depressive symptoms and on the association between current employment status and depressive symptoms, we calculated bivariate and multiple linear regression models separately for women and men.

## Results

Bivariate regression analyses indicated that, without controlling for further influences, working conditions were associated with depressive symptoms. Among men, full-time employment history was related to decreased depressive symptoms (*p* = 0.077), while part-time employment was related to higher levels of depressive symptoms (*p* = 0.065). No statistically significant relationship between employment history and depressive symptoms was found for women.

Considering only cohort affiliation, occupational education and contemporary partnership status, we found no differences between age cohorts and no effect of education on the risk of developing depressive symptoms. Being in a partnership, however, was associated with lower levels of depressive symptoms (Tables 4, 5, column 1).

**Table 4:** Depressive symptoms among male employees

	0^a^	1	2	3	4	5	6	7
Cohort affiliation								
Born 1959	Ref.	Ref.	Ref.	Ref.	Ref.	Ref.	Ref.	Ref.
Born 1965	0.69 (0.59)	0.62 (0.56)	0.64 (0.56)	0.55 (0.54)	0.58 (0.55)	0.63 (0.55)	0.65 (0.56)	0.51 (0.53)
Education—CASMIN								
Low	2.03 (1.60)	1.92 (2.11)	1.71 (2.10)	1.07 (2.02)	1.09 (2.04)	1.52 (2.18)	1.63 (2.10)	0.57 (2.06)
Middle	−0.62 (0.65)	Ref.	Ref.	Ref.	Ref.	Ref.	Ref.	Ref.
High	0.31 (0.68)	0.48 (0.65)	0.35 (0.65)	0.47 (0.65)	0.39 (0.64)	1.01 (0.65)	0.30 (0.67)	0.87 (0.65)
Partnership status								
No partner	Ref.	Ref.	Ref.	Ref.	Ref.	Ref.	Ref.	Ref.
Partner	−4.71*** (0.91)	−4.67*** (1.25)	−4.50*** (1.24)	−4.52*** (1.21)	−4.38*** (1.21)	−4.25*** (1.25)	−4.51*** (1.24)	−4.25*** (1.21)
Current employment status								
Regular full-time	−3.20* (1.32)		Ref.	Ref.	Ref.	Ref.	Ref.	Ref.
Regular part-time	4.82** (1.65)		4.10^+^ (2.17)	3.17 (2.14)	4.26* (2.16)	4.13^+^ (2.16)	3.50 (2.18)	3.05 (2.13)
Marginal	0.32 (2.13)		0.15 (2.69)	−0.39 (2.62)	0.03 (2.55)	−0.47 (2.71)	0.04 (3.12)	−1.11 (2.86)
Working conditions								
Job insecurity^b^	6.82*** (1.14)			6.61*** (1.15)				5.09*** (1.14)
Quality of leadership^c^	−2.88*** (0.33)				−2.85*** (0.33)			−2.28*** (0.35)
Influence at work^d^	−1.45*** (0.26)					−1.46*** (0.27)		−0.97*** (0.27)
Employment history								
Mostly full-time	−1.90^+^ (1.25)						Ref.	Ref.
Mostly part-time	3.32^+^ (2.02)						1.79 (2.00)	1.27 (1.95)
Mostly other states	1.05 (1.29)						0.19 (1.53)	0.39 (1.40)
*R* ^2^		0.015	0.018	0.043	0.058	0.033	0.019	0.079

Multiple linear regression analyses (*n* = 1,933)

Unstandardised regression coefficients (cluster-robust SE); ^+^ *p* < 0.1, * *p* < 0.05, ** *p* < 0.01, *** *p* < 0.001

^a^Model 0: bivariate regression

^b^Dummy variable: job insecurity 1 yes, 0 no

^c^Range of answers [quality of leadership ranged between 5 (high quality of leadership) and 1 (low quality of leadership)]

^d^Range of answers [influence at work ranged between 5 (high influence at work) and 1 (low influence at work)]

**Table 5:** Depressive symptoms among female employees

	0^a^	1	2	3	4	5	6	7
Cohort affiliation								
Born 1959	Ref.	Ref.	Ref.	Ref.	Ref.	Ref.	Ref.	Ref.
Born 1965	0.09 (0.59)	0.12 (0.58)	0.12 (0.58)	0.07 (0.57)	−0.01 (0.57)	0.06 (0.57)	0.24 (0.57)	0.02 (0.57)
Education—CASMIN								
Low	2.59^+^ (1.50)	2.35 (−1.47)	2.08 (1.49)	1.83 (1.50)	2.23 (1.43)	1.84 (1.48)	2.12^+^ (1.49)	1.88 (1.43)
Middle	−1.15^+^ (0.65)	Ref.	Ref.	Ref.	Ref.	Ref.	Ref.	Ref.
High	0.55 (0.71)	0.75 (0.72)	0.90 (0.71)	0.46 (0.72)	0.69 (0.73)	1.41* (0.71)	0.96 (0.71)	0.73 (0.73)
Partnership status								
No partner	Ref.	Ref.	Ref.	Ref.	Ref.	Ref.	Ref.	Ref.
Partner	−4.68*** (0.94)	−4.60*** (0.94)	−4.87*** (0.93)	−4.57*** (0.91)	−4.55*** (0.92)	−4.68*** (0.91)	−4.83*** (0.93)	−4.24*** (0.89)
Current employment status								
Regular full-time	−0.66 (0.68)		Ref.	Ref.	Ref.	Ref.	Ref.	Ref.
Regular part-time	0.20 (0.65)		0.83 (0.68)	0.77 (0.67)	1.15^+^ (0.65)	0.54 (0.68)	1.38^+^ (0.74)	1.35^+^ (0.71)
Marginal	1.40 (1.11)		2.35* (1.18)	1.98^+^ (1.16)	3.04** (1.15)	1.77 (1.18)	4.05** (1.38)	3.56** (1.35)
Working conditions								
Job insecurity^b^	7.83*** (0.93)			7.46*** (0.92)				5.71*** (0.86)
Quality of leadership^c^	−3.23*** (0.35)				−3.23*** (0.34)			−2.62*** (0.33)
Influence at work^d^	−1.61*** (0.29)					−1.56*** (0.29)		−0.92** (0.29)
Employment history								
Mostly full-time	0.46 (0.59)						Ref.	Ref.
Mostly part-time	0.19 (0.65)						−0.60 (0.71)	−0.61 (0.67)
Mostly other states	−0.94 (0.73)						−2.35** (0.88)	−1.79* (0.88)
*R* ^2^		0.015	0.018	0.042	0.064	0.032	0.021	0.084

Multiple linear regression analyses (*n* = 2,148)

Unstandardised regression coefficients (cluster-robust SE); ^+^ *p* < 0.1, * *p* < 0.05, ** *p* < 0.01, *** *p* < 0.001

^a^Model 0: Bivariate regression

^b^Dummy variable: job insecurity 1 yes, 0 no

^c^Range of answers [quality of leadership ranged between 5 (high quality of leadership) and 1 (low quality of leadership)]

^d^Range of answers [influence at work ranged between 5 (high influence at work) and 1 (low influence at work)]

In the second step, we investigated whether depressive symptoms were associated with current employment status (Tables 4, 5, column 2). Depressive symptoms were elevated for men engaged in regular part-time employment compared to those engaged in regular full-time employment (*p* value 0.060). Depressive symptoms were elevated for women engaged in marginal employment but not for women engaged in regular part-time employment, compared to those engaged in regular full-time employment.

We further investigated whether associations between current employment status and depressive symptoms were confounded by either current working conditions (job insecurity, quality of leadership and influence at work; Tables 4, 5, columns 3–5) or previous employment history (mostly regular full-time, mostly regular part-time and mostly other states; Tables 4, 5, column 6).

Among men, the association between current regular part-time employment and depressive symptoms decreased when current job insecurity was included in the model and increased when we adjusted for leadership quality or influence at work. In these analyses, job insecurity was positively associated with depressive symptoms; both quality of leadership and influence at work were negatively associated with depressive symptoms. It should be noted that the variance explained (*R* ^2^) increased when current working conditions, especially leadership quality, were included in the model. Additionally, depressive symptoms were insignificantly elevated with past regular part-time employment. Furthermore, the association between current regular part-time employment and depression decreased when previous part-time work was included.

Among women, the association between current marginal employment and depressive symptoms decreased when controlling for job insecurity or current influence at work and increased when current leadership quality was included in the model. In addition, the explained variance increased when current working conditions (job insecurity, leadership quality or influence at work) were included in the model. Like men, women also showed more depressive symptoms with higher job insecurity and fewer depressive symptoms in the presence of higher quality of leadership and higher levels of influence at work. However, depressive symptoms were lower for women with an employment history mostly dominated by employment states other than regular part-time and full-time, in comparison with employment history mostly dominated by full-time employment. Controlling for employment history, we found that current part-time and marginally employed women showed more depressive symptoms than women currently working full-time.

When including all variables into one model, we found persisting relationships between current working conditions and depressive symptoms among men and women (Tables 4, 5, column 7). For men, we found no statistically significant relationship between current employment status or previous employment history and depressive symptoms. For women, we still found that those who were part-time or marginally employed were more likely to show depressive symptoms in comparison with full-time working women. For women with an employment history dominated by states other than full-time or part-time work, we found slightly less depressive symptoms.

## Discussion

In the present study, the associations between current employment status and depressive symptoms appear to be mediated by both current working conditions and previous employment history. Depressive symptoms were elevated among men in regular part-time employment and women in marginal employment. Among men, some of the associations between depressive symptoms and current part-time employment were likely to be explained by previous part-time work. Also, job insecurity among men seems to explain part of the relationship between part-time employment and depressive symptoms. Among women, the statistically significant associations between current part-time and marginal employment and depressive symptoms increased after controlling for previous work history. In contrast, women with long histories of minor employment are more likely to have lower levels of depressive symptoms. It might be anticipated that among women, employment trajectory is less related to depressive symptoms. One reason might be that women identify less with their work role, but more often with their family role (Cinamon and Yisrael 2002) and thus are possibly more home-centred in their preferences (Hakim 2002). In this sense, the increased level of depressive symptoms among women with full-time employment trajectories might indicate the female double burden of reconciling full-time employment with their household situation as well as the caring demands placed on them and the resulting role conflicts (Chandola et al. 2004).

Considering working conditions, the results indicate that the association between marginal employment and depressive symptoms among women seems to be mediated by the level of influence at work. Having more influence at work weakened the negative association between marginal employment and depressive symptoms. This might be interpreted as indicating that autonomy at the work place decreases the risk of developing depressive symptoms or as indicating that individuals with depressive symptoms work in positions with less autonomy. Previous findings show, for example, that autonomy at work is positively related to well-being as well as to job control or to psychological health (Theorell et al. 1998; ter Doest and de Jonge 2006; Huang et al. 2012).

However, among women, the association between marginal employment and depressive symptoms increased when quality of leadership was controlled for. This hints at a positive relationship between female marginal employment and quality of leadership that was also found in bivariate correlation analysis (table not shown) and that additionally goes along with a suppressing effect of marginal employment within multiple regression analysis. Marginally employed women experienced better quality of leadership, which in itself was associated with decreased depressive symptoms. So, we may not expect marginal employment to be related to poorer working environment in all respects.

It is striking that the explained variance in the models tested was similar for both genders, with leadership quality showing the highest explained variance, followed by the models that included job insecurity and influence at work. In addition, the association between working conditions and depressive symptoms was the strongest with job insecurity and less strong with leadership quality and the weakest with work influence.

A further look at the correlation between current full-time employment and job insecurity, influence at work and supervisor support hints at similarities to Danish findings by Rugulies et al. (2008): Job insecurity predicts development of severe depressive symptoms among men, whereas among women, low influence at work and low supervisor support predict the development of depressive symptoms. In the present paper, we found the same pattern of correlation of these psychosocial risk factors and depressive symptoms among the middle-aged German working population.

### Strengths and limitations

The strengths of the present study are that it (a) relied on a representative sample of two cohorts (1959 and 1965) of employees in Germany, (b) had retrospective information on employment biographies, (c) used validated measures of three psychosocial factors [quality of leadership (Pejtersen et al. 2010), influence at work (Pejtersen et al. 2010) and job insecurity (Siegrist et al. 2012)] and (d) controlled for relevant potential confounders, namely vocational education and partnership status. Regarding point (a), the study analysed a heterogeneous population of employees, where the effect of age was fixed by choosing two birth cohorts (1959 and 1965) of the German baby boom generation. Regarding point (b), employment register information was used to study the relationship between employment history and health. Using register information is beneficial to avoid recall or reporting problems.

However, some limitations of the study remain to be addressed. (a) The study is—apart from the retrospective information on employment histories—cross-sectional in terms of health and working conditions. The study does not have information on previous depressive symptoms; therefore, we were not able to determine to what extent changes in employment status resulted from depressive symptoms or to what extent changes in depressive symptoms resulted from employment status (Fig. 1b). The employment biography data do not contain information on employment contracts (fixed term vs. open-ended) or periods of sickness and are limited to employment subject to social security contributions.

### Comparison with other studies

The present study confirms earlier German findings (Thielen and Kroll 2013) showing that employees in minor employment (i.e. with comparatively low income and reduced social security contributions) are more likely to show depressive symptoms than people in regular employment. The study also confirmed that employment history is more strongly related to depressive symptoms among men than among women. The study confirms the Canadian findings that some of the effects of employment on health can be mediated by specific working conditions, such as the experience of job insecurity (Scott-Marshall and Tompa 2011). The present study showed, however, that sometimes mediation can suppress the effect of employment, such as in the example of quality of leadership and marginal employment among women. Furthermore, the present study has confirmed a Danish study, finding that—among a range of psychosocial factors—job insecurity, low influence and poor quality of leadership were associated with the subsequent development of depressive symptoms (Rugulies et al. 2008).

### Future research

To determine why depressive symptoms are associated with male part-time work and female marginal employment, two research activities should be encouraged. First, analyses should investigate the relative importance of selection of people into and out of various types of employment depending on their level of depressive symptoms and of the possible impact of employment status on the development of depressive symptoms (causation). Second, longitudinal analyses considering life courses should be carried out, to study effects at the beginning of the employment career (including the period of vocational education) and through adulthood as well as in the years before and after retirement age.

Our results suggest that among German women, current marginal employment is positively related to the prevalence of depressive symptoms; employment histories with mostly non-standard employment, however, are negatively related to depressive symptoms. This relationship should be investigated further, and a better description of those women is needed with regard to family situation. It might be anticipated that factors concerning private life histories are more relevant for mental well-being among women than employment trajectory.

Finally, further aspects of part-time employment and marginal employment—and subjective indicators hereof—should be examined, which might explain elevated depressive symptoms. However, this effect is not unidirectional: reduced working time can result in negative effects in some populations and positive effects in other populations (Barnett and Gareis 2000; Gareis and Barnett 2002; Herold and Waldron 1985).

## Footnotes

1 Only employed persons currently subject to social security contributions are included in the lidA study, which means that self-employed and civil servants are not included.
